# The Influence of eHealth Stress Management Interventions on Psychological Health Parameters in Patients With Cardiovascular Disease: Systematic Review and Meta-Analysis

**DOI:** 10.2196/67118

**Published:** 2025-06-02

**Authors:** Ouahiba El-Malahi, Darya Mohajeri, Alexander Bäuerle, Raluca Ileana Mincu, Christos Rammos, Christoph Jansen, Martin Teufel, Tienush Rassaf, Julia Lortz

**Affiliations:** 1 Department of Cardiology and Vascular Medicine, West-German Heart and Vascular Center Essen, University of Duisburg-Essen, Hufelandstr. 55, Essen, 45147, Germany, 49 201-723 849; 2 Clinic for Psychosomatic Medicine and Psychotherapy, LVR-University Hospital Essen, University of Duisburg-Essen, Essen, Germany; 3 Center for Translational Neuro-and Behavioral Sciences (C-TNBS), University of Duisburg-Essen, Essen, Germany

**Keywords:** stress management, mHealth interventions, digital health intervention, psychological well-being, eHealth, psychological health, mental health, cardiovascular disease, CVD, heart, systematic review, meta-analysis, chronic stress, anxiety, depression, cardiovascular condition, mobile health

## Abstract

**Background:**

Chronic stress is a critical factor influencing both physical and mental health. It can weaken the immune system, affect cardiovascular health, and lower quality of life, often leading to psychological disorders like anxiety and depression.

**Objective:**

This study aims to evaluate the effectiveness of eHealth stress management interventions on psychological health parameters, specifically anxiety, depression, stress, and quality of life in patients with cardiovascular disease (CVD).

**Methods:**

A comprehensive search was conducted across several databases, including the Cochrane Library, APA PsycInfo, Web of Science, PubMed, Embase, and clinical trial registers. Randomized controlled trials assessing the impact of eHealth stress management interventions, namely internet-based cognitive behavioral therapy (CBT), telephone-delivered CBT, internet-based stress management training, or telephone-delivered stress management training, on the specified psychological outcomes in patients with CVD were included. The control group comprised no intervention, a waitlist, (enhanced) usual care, or a web-based intervention not focusing on stress management. To evaluate potential bias, the Risk-of-Bias 2 tool was applied. A random-effects meta-analysis was performed using standard mean difference (SMD) as the effect size, with a sensitivity analysis using mean difference (MD).

**Results:**

A total of 6 randomized controlled studies were considered in the meta-analysis. In 5 studies internet-based CBT interventions were examined, while one study used an eHealth intervention based on a CBT approach. The control groups received either usual care, were placed on a waitlist, or participated in a web-based discussion forum. After the intervention period, which ranged from 8 weeks to 6 months, a significant reduction in depressive symptoms (SMD=−0.46, MD=−2.33; *P*<.001), as assessed by the Patient Health Questionnaire-9, was observed in the intervention group compared with the control group. Mental health–related quality of life, assessed by the subscale of the 12-Item Short-Form Health Survey, showed significant improvement (SMD=0.38, MD=3.89; *P*<.001) in the intervention group in comparison to the control group following the intervention period.

**Conclusions:**

The meta-analysis demonstrates that eHealth stress management interventions substantially improve psychological health parameters in patients with CVD. Given the significant positive impact, health care providers should consider integrating eHealth stress management programs into standard care for patients with CVD. These programs can be a valuable tool in mitigating the psychological burdens associated with chronic cardiovascular conditions, ultimately improving overall patient outcomes and quality of life.

## Introduction

Stress is defined as any internal or external factor that may induce biological responses. Depending on the duration of stress exposure, this condition can lead to various negative effects on both psychological and physiological well-being. While acute stress is often characterized by symptoms of the sympathetic nervous system, such as accelerated heart rate, chronic stress can cause long-term hormonal and cellular changes and weaken the immune system [1]. This makes individuals more susceptible to infectious diseases and negatively impacts cardiovascular health by contributing to arterial hypertension and promoting proinflammatory processes in the vessel endothelium through the release of cytokines and hormones [2]. Persistent stress diminishes quality of life over time and is linked to a higher prevalence of mental health disorders such as depression and anxiety [3,4]. Since stress may promote the development of depression and anxiety, these disorders are also considered important risk factors for the development or worsening of cardiovascular health issues [5]. Among other things, psychological stress can lead to the adoption of unhealthy lifestyle behaviors, that are known as potential risk factors for the development of cardiac conditions or decrease the activity in coronary arteries, which may result in the development of a myocardial infarction [1]. Reducing stress can improve mental health disorders and lead to better outcomes for individuals with cardiovascular conditions [6].

Stress reduction strategies vary based on the type and severity of stress and cardiovascular disease (CVD). Some attempts target physical activity as the main stress-reducing technique, while other measures steer for psychotherapy and overall relaxing techniques [7]. In addition to these traditional approaches, group-based stress management programs are also regarded as successful strategies, particularly distinguished within the framework of cardiac rehabilitation [8]. However, seeking help can be challenging for patients, making digital interventions indispensable in terms of overcoming barriers. These programs can include web-based mindfulness-based stress reduction, which has been shown to significantly reduce stress-related conditions [9,10]. Digital therapy options also include web-based cognitive behavioral therapy (CBT), which notably improves stress management by offering the flexibility of accessing treatment from any location, thereby increasing its availability [11,12].

Due to the multifactorial nature of stress and its associated conditions, there is no single method for measurement. For typical physiological stress, vital parameters such as heart rate, blood pressure, and respiratory rate may change. Laboratory tests may show increased levels of stress biomarkers, such as cortisol, catecholamines, glucose, and C-reactive protein [13,14]. The psychological effects of stress are assessed through standardized questionnaires. A well-established method is the Perceived Stress Scale, which allows a qualitative analysis of individual stress perception in daily life situations [15]. Additionally, there are also scales available that assess psychological well-being under consideration of anxiety, depression, and stress simultaneously. Notable examples include the Depression Anxiety Stress Scales-21 (DASS-21) or the Hospital Anxiety and Depression Scale (HADS) [16,17].

The link between stress and mental health disorders, as well as between stress and CVD, is well-documented [18-20]. Reducing stress is crucial for cardiovascular risk. Since mental health disorders often create barriers to seeking professional help, eHealth interventions can provide more accessible options for stress management. Our systematic review and meta-analysis aimed to investigate the impact of eHealth stress management interventions on psychological health parameters, specifically focusing on anxiety, depression, stress, and quality of life in patients with CVD.

## Methods

### General Information

The “Preferred Reporting Items for Systematic Reviews and Meta-Analyses (PRISMA)” guideline was followed in conducting this systematic review and meta-analysis [21]. We used the “International Prospective Register of Systematic Reviews (PROSPERO)” to register our work (CRD42024495179).

### Ethical Considerations

Since only published data were used and analyzed, no ethics approval or written informed consent from the participants analyzed within the included studies was required.

### Search Process

We conducted systematic literature research on the databases Cochrane Library, APA PsycInfo, and Web of Science using only free text. On PubMed we used a combination of free text and Medical Subject Headings (MeSHs) and on Embase, we used free text combined with terms from Emtree. The keywords we have chosen for the free text search are as follows: “eHealth,” “e-Health,” “mHealth,” “telehealth,” “digital health intervention,” “mobile,” “app,” “web,” “web-based,” “online,” “phone,” “internet,” “internet-based cognitive behavioral therapy,” “web-based cognitive behavioral therapy,” “online cognitive behavioral therapy,” “digital cognitive behavioral therapy,” “cardiovascular disease,” “heart disease,” “coronary heart disease,” “coronary artery disease,” “ischemic heart disease,” “heart attack,” “heart failure,” “cardiac failure,” “acute coronary syndrome,” “myocardial infarction,” “peripheral arterial disease,” “peripheral occlusive disease,” “cardiac rehabilitation,” “secondary prevention,” “stress management,” “stress reduction,” “distress,” “stress,” “self-management,” “self-efficacy,” “quality of life,” “risk factor modification,” “risk factor reduction,” “randomized controlled study,” and “randomized controlled trial.” By using the database for MeSHs of PubMed, we were able to identify the following suitable MeSHs for our systematic literature research: “Telemedicine”[Mesh], “Mobile Applications”[Mesh], “Internet”[Mesh], “Computers[Mesh],” “Cell Phone”[Mesh], “Cognitive Behavioral Therapy”[Mesh], “Cardiology”[Mesh], “Cardiovascular Diseases”[Mesh], “Depression”[Mesh], and “Anxiety”[Mesh].

First, a combined search term was created for the systematic literature research on PubMed using the predefined keywords, MeSH, the operators “AND” as well as “OR” and the phrase search for multiple word keywords (eg, “cardiac rehabilitation”). This created search term was then converted with the “Polyglot Search Translator” for Embase [22]. Within this converted search, MeSHs were replaced by terms from Emtree, if MeSHs and terms from Emtree were not identical. Second, we have created a combined search term like the previous one but containing only keywords for a free text search. Afterward, we converted the created search term with the “Polyglot Search Translator” for the Cochrane Library, APA PsycInfo, and Web of Science [22]. In Table S1 in Multimedia Appendix 1 [22-29], we presented the used search term for each database searched.

In addition to searching the databases, we also searched the following clinical trial registers for completed and ongoing trials: the International Clinical Trials Search Portal, the International Standard Randomized Controlled Trial Number registry, the German Clinical Trials Register (GermanCTR), and ClinicalTrials.gov. The search terms used for the systematic research on all clinical trial registers are also shown in Table S1 in Multimedia Appendix 1.

### Selection Strategy

We used the literature management software EndNote (version 20.6 on Windows 11; Clarivate Analytics) to store the identified publications and clinical trial register entries, to detect and delete duplicates, and to conduct the entire study selection process (title/abstract screening and full-text screening). The study selection process started after we imported all records including the study register entries, and after all duplicates had been removed.

Inclusion criteria were created following the PICOS (Population, Intervention, Control, Outcome, Study Design) scheme. Thus, to consider a study eligible, the following criteria had to be met:

- Population: adults (at least 18 years old) having congestive heart failure, coronary artery disease or acute coronary syndrome, ischemic heart disease, or peripheral arterial disease.
- Intervention: internet-based CBT, telephone-delivered CBT, internet-based stress management training (SMT), or telephone-delivered SMT;
- Control: no intervention, waitlist, (enhanced) usual care, or a web-based intervention that does not address stress management.
- Outcome: stress or distress (assessed using stress-related questionnaires, eg, Perceived Stress Scale [15]); depression (assessed using tools such as the Patient Health Questionnaire-9 [PHQ-9], the Cardiac Depression Scale [CDS], or the Montgomery–Åsberg Depression Rating Scale-Self Assessment [MADRS-S] [30-32]); anxiety (assessed using proven scales for assessing anxiety as the Generalized Anxiety Disorder-7 [GAD-7] and the Cardiac Anxiety Questionnaire [CAQ] [33,34]); or quality of life (assessed using one of the following established questionnaires: the 36-Item Short-Form Health Survey, the 12-Item Short-Form Health Survey [SF-12], the Assessment of Quality of Life [AQoL], or the Heart Disease–Specific, Health-Related Quality of Life Questionnaire [35-38]).
- Study design: randomized controlled trial (RCT).

Exclusion criteria were defined regarding the population, intervention, outcome, and publication type as follows:

- Population: all patients examined in the study were diagnosed with atrial fibrillation, congenital heart disease, or both.
- Intervention: lifestyle interventions, telemonitoring, self-care interventions not addressing or focusing on stress management, behavioral activation, or motivational interviewing that are not delivered as part of CBT or SMT.
- Intervention period: less than 2 weeks.
- Outcome: not reported for intervention or control group after the intervention period.
- Publication type: conference abstracts of screened studies, publications without accessible abstract or full text.

Considering the inclusion and exclusion criteria, a title and abstract review was undertaken for publications as the first part of the study selection process. All reviewed publications were either directly excluded with a note or taken into consideration for a full-text screening, which then decided on study inclusion or exclusion with a note. All notes regarding the exclusion of a publication within the first or second part of the study selection process were documented in EndNote. The results of the selection process were discussed between 2 researchers.

The first part of the screening process for registered clinical trials (records from clinical trial registers) was similar to the screening process described above. The title and all available information were read and assessed for eligibility. Only eligible studies were taken into consideration for the second part of the screening process. We searched on PubMed and Google for publications linked to the study identification number. Once a match was found, the corresponding report was linked to the study in EndNote, and a full-text screening of this report was performed. In cases where we were unable to find suitable publications, we requested study reports from the contacts listed in the study register entry. No study report was requested for ongoing studies. These studies were included or excluded based on available and other researched information.

### Data Extraction and Quality Assessment

Data extraction was performed separately for eligible studies with already published results and for ongoing studies using 2 different Microsoft Excel files. Regarding the completed studies, we decided to extract data as follows: first author (last name), publication year, registration number, important information for assessing the risk of bias (study design, randomization method, blinding, and intention-to-treat-analysis), sample sizes and demographic data (age and gender distribution), investigated CVD (acute coronary syndrome, coronary artery disease, congestive heart failure, or peripheral arterial disease), number of participants with other diseases (eg, heart diseases different from the investigated diseases, diabetes, cancer), participation criteria, delivered intervention (type, period, and number of sessions), information regarding the control group, and outcome of interest including the assessment method and the results of interest measured at baseline and after the intervention period.

As far as the ongoing studies are concerned, we have decided to extract general study information (official name and registration number), inclusion criteria, study start, delivered interventions, and outcomes of interest in the Microsoft Excel file created for this purpose. All data were extracted based on study register entries or researched study protocols, for those available.

Quality assessment was performed by evaluating the risk of bias. For this purpose, we decided to use the Risk-of-Bias 2 tool that addresses the following domains: selection bias, performance bias, detection bias, attrition bias, reporting bias, and other biases [39].

### Data Synthesis

Microsoft Excel was used for data synthesis, summarizing the means and SDs for all reported mental well-being outcome parameters as follows: (1) anxiety rated with the GAD-7 and the CAQ; (2) depression rated with the CDS, the PHQ-9 and the MADRS-S; (3) anxiety/depression rated with the HADS; (4) stress rated with the DASS-21; and (5) quality of life with rated with the SF-12 and the AQoL-8D [16,17,30-37]. These values were summarized at 2 time points (baseline and postintervention) for the intervention group and for the control group.

### Meta-Analysis

A meta-analysis was performed for outcome parameters reported by at least 2 studies. In some cases (eg, CAQ-avoidance) a meta-analysis was also conducted for the subscales. Thus, the following psychological well-being outcome parameters were investigated within a meta-analysis: CAQ (CAQ-total, CAQ-avoidance, CAQ-attention, and CAQ-fear) and GAD-7 for assessing anxiety, MADRS-S and PHQ-9 for assessing depression, and SF-12 (SF-12 Physical Health and SF-12 Mental Health) for assessing quality of life. The meta-analyses for anxiety and depression were conducted based on the measurement instrument used in at least 2 studies, as most studies reported anxiety and depression using multiple scales [23-27]. For the meta-analysis of quality of life, the assessment methods used within the studies were not comparable in that the studies that used the SF-12 assessment method reported quality of life with 2 scores, the Physical Health and the Mental Health score [23,24,26], whereas the study using the AQoL-8D assessment method delivered one total score [28]. For this reason, no scale-independent meta-analysis was conducted for the psychological well-being outcome parameter anxiety, depression, or quality of life.

The conducted meta-analysis is based on a random effects model and only postinterventional values were used. All analyses were performed using standard mean difference (SMD) to indicate the effect size and *I*^2^ statistics to evaluate heterogeneity.

The sensitivity analysis was performed using a different effect size for each outcome parameter. For this, we decided to use the effect size “mean difference.” In view of the small number of studies (≤5 studies) within each meta-analysis, we decided not to conduct subgroup analyses for any outcome parameter.

All analyses were performed with the software IBM SPSS Statistics for Windows (version 29.0.0.0; International Business Machines Corporation).

## Results

### Study Selection

**Figure 1.**
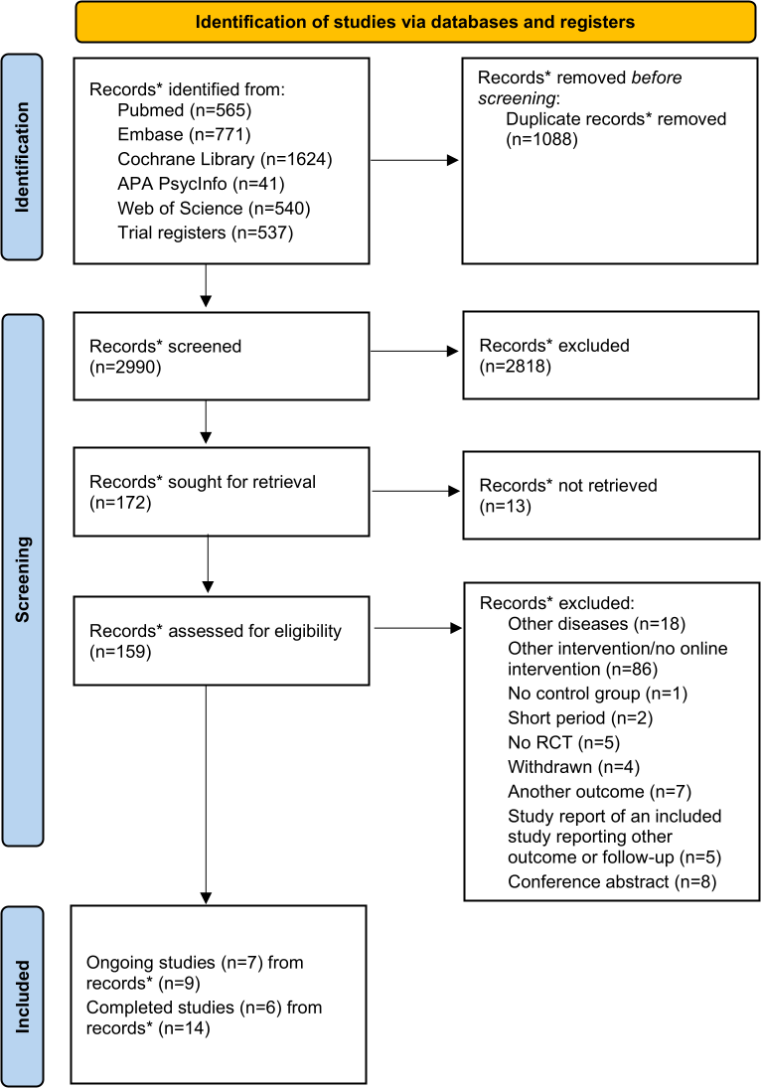
PRISMA (Preferred Reporting Items for Systematic Reviews and Meta-Analysis) flow chart providing an overview of the study selection process for the systematic review. RCT: randomized controlled trial. *The term “records” includes both various study reports as well as study registry entries or other documents that may be related to one study.

Our systematic literature search initially yielded 2990 records, including publications and clinical trial registrations. After removing duplicates, we identified a total of 7 ongoing studies [40-48] and 6 completed studies [23-29] that met our eligibility criteria and were included in the analysis. The whole study selection process is detailed in the PRISMA (Preferred Reporting Items for Systematic Reviews and Meta-Analysis) flow chart [21] in Figure 1.

### Study Characteristics

An overview of the 7 eligible and currently ongoing studies can be found in Table 1 [40-48]. The study characteristics of the 6 completed studies [23-29] are summarized in Table 2. As far as the completed studies are concerned, the total sample sizes vary ranging from 34 to 239 participants [25,28]. The highest mean age of the participants in the intervention group was 63.6 (SD 13.9) years [29], whereas the highest mean age of the participants in the control group was 64.0 (SD 12.0) years [26]. Control groups within the completed studies received usual care [23,25], participated in a web-based discussion forum [26,29], or were placed on the waiting list [24,28]. One study examined patients with atrial fibrillation in addition to patients with congestive heart failure or acute coronary syndrome. As the total number of patients with congestive heart failure and acute coronary syndrome represented the majority of patients, we decided to include this study in our systematic review and meta-analysis [26]. Depression as a physical well-being outcome of interest was reported in all 6 studies [23-26,28,29], anxiety in 5 studies [24,25,27-29], quality of life in 4 studies [23,24,26,28], and stress/distress in one study [24].

**Table 1.** Overview of currently ongoing studies investigating eHealth interventions on psychological well-being.

Study identification number	Investigated heart disease	Intervention	Control	Outcome of interest
DRKS00020824 [40,41]	Coronary artery disease and acute coronary syndrome	Telephone-delivered blended collaborative care intervention + usual care	Usual care	Anxiety or depression (HADS^a^), quality of life, and stress (PSS-4^b^)
NCT02914483 [42,43]	Myocardial infarction	Telephone-based mindfulness-based cognitive therapy	Enhanced usual care	Anxiety or depression (HADS), depression (PHQ-9^c^), quality of life (SF-12^d^), and stress (PSS-10)
NCT04172974 [44]	Ischemic heart disease	eHealth intervention “eMindYourHeart” + usual care	Usual care	Anxiety or depression (HADS and CAQ) depression (PHQ-9), quality of life (HeartQoL^e^), and stress (PSS-10)
NCT05580718 [45]	Myocardial infarction	Internet-delivered CBT^f^	Waitlist + usual care	Anxiety (CAQ^g^ and GAD-7^h^), depression (PHQ-9), stress (PSS-4), and quality of life (SF-12)
NCT05607992 [46]	Acute coronary syndrome	Internet-delivered CBT	Waitlist	Anxiety (CAQ and GAD-7), depression (PHQ-9), stress (PSS-4), and quality of life (SF-12)
NCT05846334 [47]	Ischemic heart disease	mHealth intervention “mindfulHeart”	Usual care	Anxiety (STAI^i^), depression (BDI-II^j^), stress or distress (GHQ-12^k^, PSS-10, and combined stress measure), and quality of life (SF-36)
NCT05967247 [48]	Congestive heart failure	mHealth mindfulness-based stress reduction intervention “Mindfulness in Life (Taiwan)”	Waitlist + self-management app “Heart Care Life”	Depression (PHQ-9) and quality of life (SF-12)

a HADS: Hospital Anxiety and Depression Scale.

b PSS: Perceived Stress Scale.

c PHQ-9: Patient Health Questionnaire-9.

d SF-12: 12-Item Short-Form Health Survey.

e HeartQoL: Heart Disease–Specific, Health-Related Quality of Life Questionnaire.

f CBT: cognitive behavioral therapy.

g CAQ: Cardiac Anxiety Questionnaire.

h GAD-7: Generalized Anxiety Disorder-7.

i STAI: State-Trait Anxiety Inventory.

j BDI-II: Beck Depression Inventory-II.

k GHQ-12=General health Questionnaire-12.

**Table 2.** Characteristics of completed studies investigating eHealth interventions in patients with cardiovascular disease.

Author	Year	Sample size and mean age	Main heart disease	Intervention	Control	Period	Outcome of interest
O’Neil et al [23]	2014	Intervention (size=61), 61.0 (SD 10.2) years Control (size=60), 58.9 (SD 10.7) years	Acute coronary syndrome	Tele-health intervention “MoodCare” with CBT^a^ approach	Usual care	6 months	Depression (CDS^b^ and PHQ-9^c^) and quality of life (SF-12^d^)
Bendig et al [Bibr R38][28]	2021	Intervention (size=18), none Control (size=16), none	Coronary artery disease	Internet-based CBT	Waitlist	8 weeks	Anxiety (GAD-7^e^), depression (PHQ-9), and quality of life (AQoL-8D^f^)
Schneider et al [Bibr R34][24]	2020	Intervention (size=25), 56.7 (SD 11.9) years Control (size=28), 59.3 (SD 6.9) years	Acute coronary syndrome	Internet-based CBT (“Cardiac Wellbeing Course”)	Waitlist	8 weeks	Anxiety (GAD-7 and CAQ^g^), depression (PHQ-9), stress or distress (DASS-21^h^), and quality of life (SF-12)
Norlund et al [Bibr R35][25]	2018	Intervention (size=117), 58.4 (SD 9.0) years Control (size=122), 60.8 (SD 7.8) years	Myocardial infarction	Internet-based CBT	Usual care	14 weeks	Anxiety or depression (HADS^i^), anxiety (CAQ), and depression (MADRS-S^j^)
Lundgren et al [Bibr R48][29]	2016	Intervention (size=25), 63.6 (SD 13.9) years Control (size=25), 62.3 (11.7) years	Congestive heart failure	Internet-based CBT	Web-based discussion forum	9 weeks	Anxiety (CAQ) and depression (PHQ-9)
Johansson et al^k^ [Bibr R36][26]	2019	Intervention (size=72), 61.0 (SD 13.0) years Control (size=72), 64.0 (SD 12.0) years	Coronary artery disease, congestive heart failure, atrial fibrillation	Internet-based CBT	Web-based discussion forum	9 weeks	Anxiety (GAD-7 and CAQ), depression (PHQ-9 and MADRS-S), and quality of life (SF-12)
Westas et al^k^ [Bibr R37][27]	2023	Intervention (size=72), 61.0 (SD 13.0) years Control (size=72), 64.0 (SD 12.0) years	Coronary artery disease, congestive heart failure, atrial fibrillation	Internet-based CBT	Web-based discussion forum	9 weeks	Anxiety (GAD-7 and CAQ), depression (PHQ-9 and MADRS-S), and quality of life (SF-12)

a CBT: cognitive behavioral therapy.

b CDS: Cardiac Depression Scale.

c PHQ-9: Patient Health Questionnaire-9.

d SF-12: 12-Item Short-Form Health Survey.

e GAD-7: Generalized Anxiety Disorder-7.

f AQoL-8D: Assessment of Quality of Life-8D.

g CAQ: Cardiac Anxiety Questionnaire.

h DASS-21: Depression Anxiety Stress Scales-21.

i HADS: Hospital Anxiety and Depression Scale.

j MADRS-S: Montgomery–Åsberg Depression Rating Scale-Self Assessment.

k These are two different reports from the same study.

### Risk of Bias

**Figure 2.**
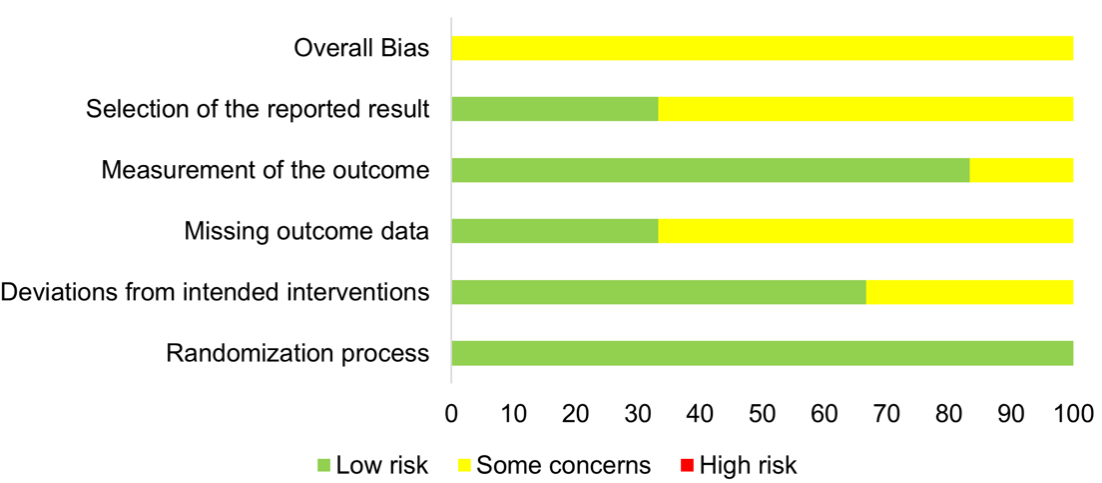
Risk of bias graph showing the findings of quality assessment for all 6 studies.

All 6 completed studies, which are summarized in Table 2, underwent assessment for potential bias. There was neither a study with a high risk of bias in any of the 5 domains nor with an overall low risk of bias as presented in Figure 2. Two studies were found to have some concerns regarding the second domain, which assessed deviations from the intended interventions [24,25]. Four studies were assessed with some concerns regarding the domain addressing missing outcome data [23,25,26,29] and the domain related to the selection of reported results [24,26,28,29]. The domain dealing with outcome measurement was evaluated in one study with some concerns [24].

### Meta-Analysis

The meta-analyses included all 6 completed studies and were conducted on the basis of the outcome parameters reported in 7 study reports [23-29]. Meta-analysis was not undertaken for outcome parameters of interest that were assessed using the following questionnaires, as they were only reported in one study each: AQoL-8D [28], HADS [25], CDS [23], and DASS-21 [24].

One study reporting CAQ values for the intervention group was excluded from the meta-analysis of the parameter CAQ due to the lack of detailed values (mean and SD) for the control group [29].

### Anxiety

#### Cardiac Anxiety Questionnaire (CAQ)

**Figure 3.**
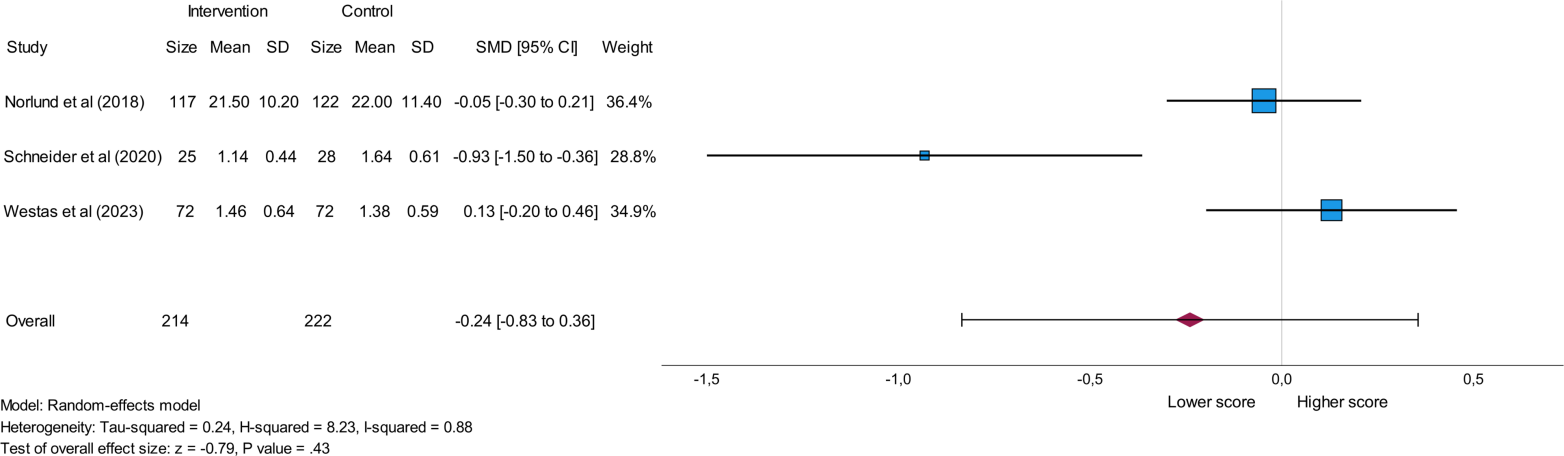
Forest plot presenting the impact of eHealth stress management interventions on the CAQ-total (Cardiac Anxiety Questionnaire) score among patients with cardiovascular disease. SMD: standard mean difference [24,25,27].

The CAQ-total score was analyzed within a meta-analysis based on 3 eligible studies and involved 214 participants in the intervention group and 222 participants in the control group. All 3 studies implemented internet-based CBT as an intervention [24,25,27]. A lower CAQ-total score in the intervention group reflecting a decrease in cardiac anxiety levels [34] was reported in 2 studies [24,25], while one study reported a lower CAQ-total score in the control group in comparison to the intervention group [27]. The analysis showed a nonsignificant result (*P*=.43) and indicated a small effect with the obtained overall effect size (SMD=−0.24). Heterogeneity between studies was high, as shown by an *I*^2^ value of 0.88, indicating considerable variability within the data. The results of this meta-analysis are presented in Figure 3.

Meta-analysis of the subscales CAQ-attention, CAQ-avoidance, and CAQ-fear included 2 studies with a total of 97 participants in the intervention group and a total of 100 participants in the control group. In both studies, internet-based CBT was used as an intervention [24,27]. None of these meta-analyses showed statistical significance (CAQ-attention, *P*=.39; CAQ-avoidance, *P*=.37; CAQ-fear, *P*=.63) as presented in Figures S1-S3 in Multimedia Appendix 1. Estimated overall effect sizes were either small (CAQ-attention, SMD=−0.42; CAQ-fear, SMD=−0.30) or very small (CAQ-avoidance, SMD=−0.13). Heterogeneity between studies was high for CAQ-attention as shown by an *I*^2^ value of 0.88 and was also indicated high for CAQ-fear with an *I*^2^ value of 0.93. Meta-analysis of CAQ-avoidance resulted in no heterogeneity as presented by an *I*^2^ value of 0.00.

#### Generalized Anxiety Disorder-7 (GAD-7)

**Figure 4.**
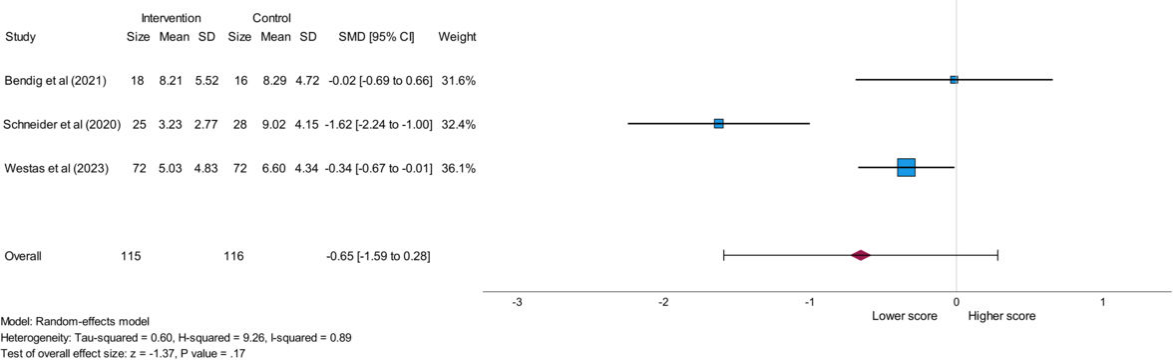
Forest plot showing the impact of eHealth stress management interventions on the GAD-7 (Generalized Anxiety Disorder-7) score in patients with cardiovascular disease. SMD: standard mean difference [24,27,28].

The outcome parameter GAD-7 was analyzed using 3 eligible studies that involved a total of 115 participants in the intervention group and a total of 116 participants in the control group. All analyzed studies used internet-based CBT as an intervention and reported a lower GAD-7 value in the intervention group compared with the control group [24,27,28] indicating reduced anxiety [33]. This meta-analysis showed a medium effect (SMD=−0.65) and a nonsignificant result (*P*=.17) with high heterogeneity, as expressed by the *I*^2^ value of 0.89 and presented in Figure 4.

### Depression

#### Montgomery–Åsberg Depression Rating Scale-Self Assessment (MADRS-S)

The meta-analysis of MADRS-S was based on 2 studies investigating a total of 189 participants in the intervention group receiving internet-based CBT and a total of 194 participants in the control group [25,26]. A lower MADRS-S value was assessed in the intervention group indicating a decrease in depressive symptoms [32] in both studies examined [25,26]. This analysis (Figure S4 in Multimedia Appendix 1) revealed an overall small effect (SMD=–0.41) with a nonsignificant value (*P*=.10) and high heterogeneity, that was characterized by an *I*^2^ value of 0.82.

#### Patient Health Questionnaire-9 (PHQ-9)

**Figure 5.**
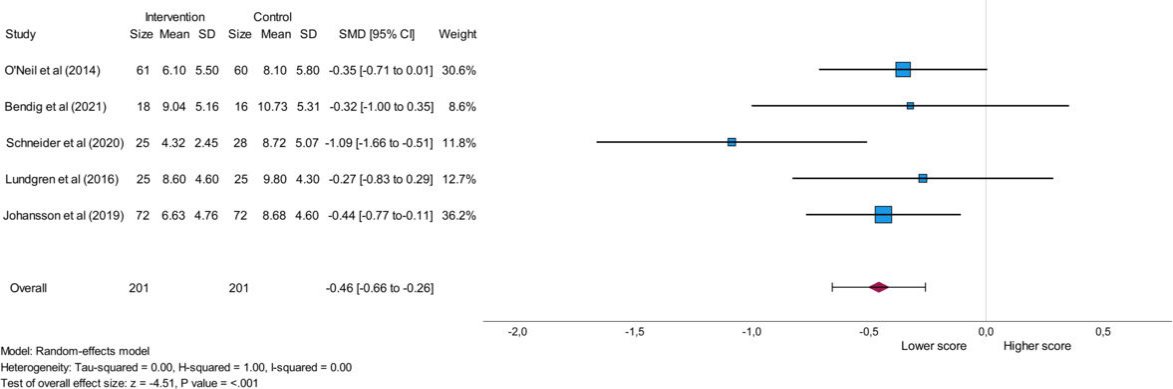
Forest plot highlighting the effect of eHealth stress management interventions on the PHQ-9 (Patient Health Questionnaire-9) score in patients with cardiovascular disease. SMD: standard mean difference [23,24,26,28,29].

The PHQ-9 outcome parameter was analyzed within a meta-analysis based on 5 studies exploring internet-based CBT or a telehealth intervention grounded in CBT. Both the intervention group and the control group consisted of a total of 201 participants each [23,24,26,28,29]. Lower PHQ-9 values in the intervention group were reported in all 5 studies compared with the control group [23,24,26,28,29] reflecting an alleviation of depressive symptoms [30]. This analysis is presented in Figure 5 and showed a significant result (*P*<.001), an overall small effect (SMD=−0.46), and no heterogeneity as expressed by an *I*^2^ value of 0.00.

### Quality of Life

**Figure 6.**
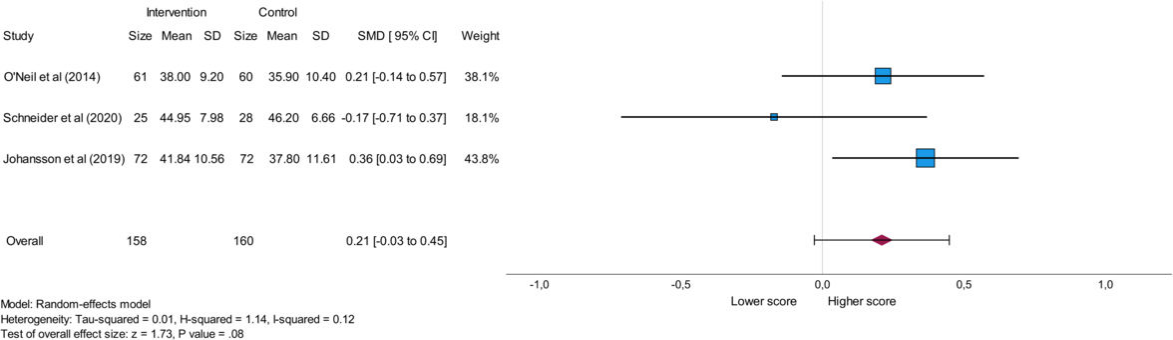
Forest plot displaying the impact of eHealth stress management interventions on the SF-12 (12-Item Short-Form Health Survey) physical health score in patients with cardiovascular disease. SMD: standard mean difference [23,24,26].

Analysis of the SF-12 score for physical health was based on 3 studies investigating a total of 158 participants in the intervention group and a total of 160 participants in the control group [23,24,26]. One of the analyzed studies reported a lower SF-12 score for physical health in the intervention group in contrast to the control group [24] suggesting poorer physical health [35], while the other studies reported higher values in the intervention group compared with the control group [23,26]. This analysis showed a nonsignificant result (*P*=.08), an overall small effect (SMD=0.21), and negligible heterogeneity characterized by the *I*^2^ value of 0.12. The results of this meta-analysis are shown in Figure 6.

**Figure 7.**
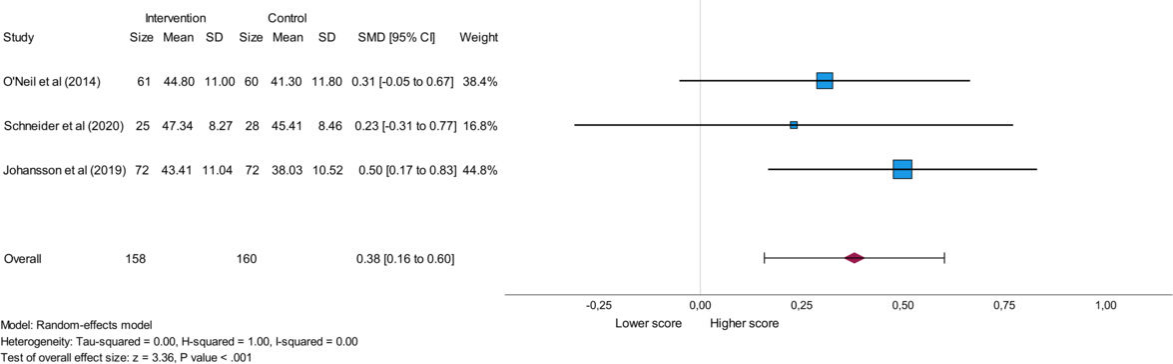
Forest plot presenting the influence of eHealth stress management interventions on the SF-12 (12-Item Short-Form Health Survey) mental health score in patients with cardiovascular disease. SMD: standard mean difference [23,24,26].

The SF-12 score for mental health was analyzed based on the same 3 studies as the SF-12 score for physical health and also included a total of 158 participants in the intervention group and a total of 160 participants in the control group [23,24,26]. All 3 studies reported higher values in the intervention group [23,24,26] indicating improved psychological well-being [35]. This analysis, shown in Figure 7, yielded a significant value (*P*<.001) with an overall small effect size (SMD=0.38) and no heterogeneity that was reflected in an *I*^2^ value of 0.00.

### Sensitivity Analysis

The sensitivity analysis conducted on the outcome parameters linked to anxiety all resulted in nonsignificant findings. Two studies each were involved in the analysis of the CAQ-total score, the CAQ-attention score, the CAQ-avoidance score, and the CAQ-anxiety score [24,27], while the sensitivity analysis of the GAD-7 score included a total of 3 studies [24,27,28].

Regarding the outcome parameters measuring depression, the sensitivity analysis of the PHQ-9 score that included 5 studies [23,24,26,28,29] showed significance (*P*<.001), while the analysis of the outcome parameter MADRS-S, which was based on 2 studies [25,26] yielded a non-significant result.

Sensitivity analysis of the SF-12 Physical Health score and the SF-12 Mental Health score, which express quality of life, included 3 studies each [23,24,26]. The SF-12 Mental Health score yielded a significant result (*P*<.001), while the SF-12 Physical Health score yielded a nonsignificant value.

The mean difference and its CI, the SE, the *P* value, and the *I*^2^ values expressing heterogeneity determined in the sensitivity analysis for each outcome parameter are presented in Table S2 in Multimedia Appendix 1.

## Discussion

### Principal Findings

This systematic review and meta-analysis examined the effects of eHealth stress management interventions on psychological health parameters, including anxiety, depression, and quality of life, as measured by psychometric instruments. Significant postinterventional differences between the intervention and control groups were found for depression, indicated by the PHQ-9 score, and for quality of life, measured by the SF-12 Mental Health score. No significant effects were observed for anxiety, as measured by the CAQ or GAD-7 score, nor for depression, as measured by the MADRS-S, nor for quality of life, as expressed by the SF-12 Physical Health score. It is important to note that the analysis of the MADRS-S included fewer studies [25,26] compared with the PHQ-9 analysis [23,24,26,28,29]. Aside from this, the result regarding the MADRS-S showed a high level of heterogeneity, which could potentially be attributed to differing group sizes or intervention periods within the studies [25,26]. Since no heterogeneity was observed in the analysis of the PHQ-9 score, it can be assumed that the overall effect regarding depression, as measured by the MADRS-S, was negatively influenced by the high heterogeneity. This is likely not the case for the PHQ-9, due to the absence of heterogeneity. The analysis of SF-12 Physical Health and SF-12 Mental Health was conducted using the same set of studies [23,24,26]. Our meta-analysis finding that internet-based CBT has no significant effect on the SF-12 Physical Health score is consistent with the findings of a previous meta-analysis that investigated the impact of CBT in patients with CVD [49].

### Comparison to Prior Work

Since all conducted analyses included studies that investigated internet-based CBT [24-29] or an eHealth intervention with a CBT approach [23], and both the PHQ-9 score and the SF-12 Mental Health score showed significant changes with the intervention, a positive effect can be attributed to internet-based CBT. Previous meta-analyses have also demonstrated that CBT can reduce depression [50,51] and improve mental health-related quality of life in patients with CVD compared with a control group [49]. Another already published meta-analysis, which included studies on both CBT and internet-based CBT, showed positive effects on anxiety, depression, stress, and quality of life in patients with cardiac conditions [52]. These findings align well with results from a prior research article examining the impact of internet-based CBT on patients experiencing increased stress and stress-related disorders [53]. We were unable to confirm these results for anxiety and stress in relation to internet-based CBT. Our analyses for anxiety did not show significant results, and the number of studies that assessed stress using the same assessment method was insufficient to conduct a meta-analysis. However, it is important to note that we examined the effects of eHealth stress interventions on anxiety, depression, and quality of life depending on the measurement method used. We identified differences between the 2 groups in terms of anxiety levels after the treatment period using the estimated overall effect sizes. In the sensitivity analysis, the same outcome parameters yielded significant results on depression measured by the PHQ-9 score and quality of life measured by the SF-12 Mental Health score. We conclude that both effect sizes lead to comparable results.

Our research highlights the use of internet-based CBT for enhancing psychological health parameters in patients with CVD. Recent studies investigated its application and impact on psychological outcome parameters in patients with other diseases [54,55]. Research on effectively reducing stress, depression, and anxiety is important, especially since the success of treatment and adherence can be negatively impacted, such as among patients with cancer [56].

### Future Directions

In addition to internet-based CBT, other internet-based and computer-based stress management interventions have shown potential to positively impact psychological health parameters by reducing stress, anxiety, and depression, as demonstrated in a previous meta-analysis [57]. Mindfulness-based interventions via the web generally demonstrate stress-reducing effects, as evidenced by a conducted meta-analysis involving patients without diagnosed psychological or physical illnesses [58]. Therefore, further exploration of these interventions beyond internet-based CBT, as indicated by ongoing studies included in this work [40,41,44,47,48], can yield additional insights in this field. It could be also beneficial to explore the implementation of web-based exercise programs for stress reduction in the secondary prevention of heart disease. Physical activity has the potential to significantly enhance heart health by improving parameters of heart rate variability, which may be adversely affected by stress [59]. Mind-body exercises such as yoga but also interventions like HRV-biofeedback have been shown to have positive effects on stress reduction in individuals with CVD, as indicated by recent research [60]. Internet-based approaches or variations of these or other effective stress management interventions should therefore be considered, as several studies have demonstrated the positive impact of eHealth interventions on patients with CVD [61] and high patient acceptance for eHealth cardiac rehabilitation programs in secondary prevention is given [62].

### Limitations

This meta-analysis faced some limitations. First, we decided to include a study in which not all participants experienced atrial fibrillation, though a considerable number of them did. The results reported in that study cannot be attributed exclusively to patients with coronary artery disease or congestive heart failure which represented the majority of the patient population examined within the study [26]. For future research, it would therefore be of interest to examine patients with atrial fibrillation only, to better understand the impact of eHealth stress management interventions specifically in relation to this condition. Second, high heterogeneity between the studies was also found within some analyses. The causes of the heterogeneity could be attributed to the number of study participants, the duration of the intervention period, or the differences regarding the control group. However, due to the small number of studies and the resulting lack of subgroup analysis, the cause of heterogeneity could not be determined. Third, all analyses conducted included less than 15 studies which is due to the limited number of available and comparable studies. This means that the results presented should be interpreted with caution and that further research is needed in this area. Fourth, important supplementary information, such as the total number of sessions and the number of sessions completed by all participants, was not considered in our review, as it proved difficult to compare this information meaningfully due to the differing intervention durations or the varied structure of the intervention modules. This indicates that future research should also focus on this aspect to draw meaningful conclusions from the quantity and frequency of the sessions. Fifth, since we had only one already completed study examining the impact of an eHealth stress management intervention on stress [24], we were unable to assess its influence on stress within the meta-analysis. More studies are needed that examine the effects of eHealth stress management methods on stress in patients with CVD.

### Conclusions

Overall, this meta-analysis validates the positive influence of eHealth stress management interventions on psychological health parameters in patients with CVD and marks the importance of further clinical approaches for digital health strategies.

## Supplementary material

10.2196/67118 Multimedia Appendix 1 Supplementary material including search strategy, meta-analysis, and sensitivity analysis.

10.2196/67118 Checklist 1 PRISMA (Preferred Reporting Items for Systematic Reviews and Meta-Analysis) checklist.

## Abbreviations

AQoL: Assessment of Quality of Life
CAQ: Cardiac Anxiety Questionnaire
CBT: cognitive behavioral therapy
CDS: Cardiac Depression Scale
CVD: cardiovascular disease
DASS-21: Depression Anxiety Stress Scales-21
GAD-7: Generalized Anxiety Disorder-7
HADS: Hospital Anxiety and Depression Scale
MADRS-S: Montgomery–Åsberg Depression Rating Scale-Self Assessment
MD: mean difference
MeSH: Medical Subject Heading
PHQ-9: Patient Health Questionnaire-9
PICOS: Population, Intervention, Control, Outcome, Study Design
PRISMA: Preferred Reporting Items for Systematic Reviews and Meta-Analysis
PROSPERO: International Prospective Register of Systematic Reviews
SF-12: 12-Item Short-Form Health Survey
SMD: standard mean difference
SMT: stress management training
