# Supplementary material for: The Influence of eHealth Stress Management Interventions on Psychological Health Parameters in Patients With Cardiovascular Disease: Systematic Review and Meta-Analysis
Source: J Med Internet Res. 2025 Jun 2;27:e67118. doi: 10.2196/67118 (PMC12148253; doi:10.2196/67118)
Supplement: Multimedia Appendix 1 [file jmir-v27-e67118-s001.pdf]

The influence of eHealth stress management interventions on psychological health parameters in patients with cardiovascular disease: A systematic review and meta-analysis

SUPPLEMENTARY MATERIAL

Ouahiba El-Malahi[a], Darya Mohajeri[a], Alexander Bäuerle[b,c], Raluca Mincu[a], Christos Rammos[a], Christoph Jansen[b,c], Martin Teufel[b,c], Tienush Rassaf[a], Julia Lortz[a]

[a] Department of Cardiology and Vascular Medicine, West-German Heart and Vascular Center Essen, University of Duisburg-Essen, Hufelandstr. 55, 45147 Essen, Germany

[b] Clinic for Psychosomatic Medicine and Psychotherapy, LVR-University Hospital Essen, University of Duisburg-Essen, Virchowstr. 174, 45147 Essen, Germany

[c] Center for Translational Neuro-and Behavioral Sciences (C-TNBS), University of Duisburg-Essen, Essen, Germany

Index

Methods 2

Search strategy 2

Results 7

Meta-analysis 7

Sensitivity analysis 9

References 10

Methods

Search strategy

Table S1. Overview of the databases searched and the search terms used

#	Source and date	Search term	Hits
**1**	Pubmed 15.11.2023	(eHealth[tiab] OR e-Health[tiab] OR mHealth[tiab] OR telehealth[tiab] OR "digital health intervention"[tiab] OR mobile[tiab] OR app[tiab] OR web[tiab] OR web-based[tiab] OR online[tiab] OR phone[tiab] OR internet[tiab] OR Telemedicine[Mesh] OR "Mobile Applications"[Mesh] OR Internet[Mesh] OR Computers[Mesh] OR "Cell Phone"[Mesh] OR "internet-based cognitive behavioral therapy"[tiab] OR "web-based cognitive behavioral therapy"[tiab] OR "online cognitive behavioral therapy"[tiab] OR "digital cognitive behavioral therapy"[tiab] OR "Cognitive Behavioral Therapy"[Mesh]) AND ("cardiovascular disease"[tiab] OR "heart disease"[tiab] OR "coronary heart disease"[tiab] OR "coronary artery disease"[tiab] OR "ischemic heart disease"[tiab] OR "heart attack"[tiab] OR "heart failure"[tiab] OR "cardiac failure"[tiab] OR "acute coronary syndrome"[tiab] OR "myocardial infarction"[tiab] OR "peripheral arterial disease"[tiab] OR "peripheral occlusive disease"[tiab] OR Cardiology[Mesh] OR "Cardiovascular Diseases"[Mesh]) AND ("cardiac rehabilitation"[tiab] OR "secondary prevention"[tiab] OR "stress management"[tiab] OR "stress reduction"[tiab] OR distress[tiab] OR stress[tiab] OR self-management[tiab] OR self-efficacy[tiab] OR "quality of life"[tiab] OR "risk factor modification"[tiab] OR "risk factor reduction"[tiab] OR Depression[Mesh] OR Anxiety[Mesh]) AND ("randomized controlled study"[tiab] OR "randomized controlled trial"[tiab])	565
**2**	Embase 15.11.2023	(eHealth:ti,ab OR e-Health:ti,ab OR mHealth:ti,ab OR telehealth:ti,ab OR 'digital health intervention':ti,ab OR	771

		mobile:ti,ab OR app:ti,ab OR web:ti,ab OR web-based:ti,ab OR online:ti,ab OR phone:ti,ab OR internet:ti,ab OR 'internet-based cognitive behavioral therapy':ti,ab OR 'web-based cognitive behavioral therapy':ti,ab OR 'online cognitive behavioral therapy':ti,ab OR 'digital cognitive behavioral therapy':ti,ab OR telemedicine/exp OR 'mobile application'/exp OR Internet/exp OR computer/exp OR 'cognitive behavioral therapy'/exp) AND ('cardiovascular disease':ti,ab OR 'heart disease':ti,ab OR 'coronary heart disease':ti,ab OR 'coronary artery disease':ti,ab OR 'ischemic heart disease':ti,ab OR 'heart attack':ti,ab OR 'heart failure':ti,ab OR 'cardiac failure':ti,ab OR 'acute coronary syndrome':ti,ab OR 'myocardial infarction':ti,ab OR 'peripheral arterial disease':ti,ab OR 'peripheral occlusive disease':ti,ab OR cardiology/exp OR 'cardiovascular disease'/exp) AND ('cardiac rehabilitation':ti,ab OR 'secondary prevention':ti,ab OR 'stress management':ti,ab OR 'stress reduction':ti,ab OR distress:ti,ab OR stress:ti,ab OR self-management:ti,ab OR self-efficacy:ti,ab OR 'quality of life':ti,ab OR 'risk factor modification':ti,ab OR 'risk factor reduction':ti,ab OR depression/exp OR anxiety/exp) AND ('randomized controlled study':ti,ab OR 'randomized controlled trial':ti,ab)<br><br>*(Polyglot [1] was used to convert the search term and this search term contained terms from Emtree)*	
**3**	Cochrane Library 15.11.2023	(eHealth:ti,ab OR e-Health:ti,ab OR mHealth:ti,ab OR telehealth:ti,ab OR "digital health intervention":ti,ab OR mobile:ti,ab OR app:ti,ab OR web:ti,ab OR web-based:ti,ab OR online:ti,ab OR phone:ti,ab OR internet:ti,ab OR "internet-based cognitive behavioral therapy":ti,ab OR "web-based cognitive behavioral therapy":ti,ab OR "online cognitive behavioral therapy":ti,ab OR "digital cognitive behavioral therapy":ti,ab) AND ("cardiovascular disease":ti,ab OR "heart disease":ti,ab OR "coronary heart disease":ti,ab OR "coronary artery disease":ti,ab OR "ischemic heart disease":ti,ab OR "heart attack":ti,ab OR	1624

		"heart failure":ti,ab OR "cardiac failure":ti,ab OR "acute coronary syndrome":ti,ab OR "myocardial infarction":ti,ab OR "peripheral arterial disease":ti,ab OR "peripheral occlusive disease":ti,ab) AND ("cardiac rehabilitation":ti,ab OR "secondary prevention":ti,ab OR "stress management":ti,ab OR "stress reduction":ti,ab OR distress:ti,ab OR stress:ti,ab OR self-management:ti,ab OR self-efficacy:ti,ab OR "quality of life":ti,ab OR "risk factor modification":ti,ab OR "risk factor reduction":ti,ab)<br>*(Polyglot [1] was used to convert the search term)*	
**4**	APA PsycInfo<br>15.11.2023	(eHealth.ti,ab. OR e-Health.ti,ab. OR mHealth.ti,ab. OR telehealth.ti,ab. OR "digital health intervention".ti,ab. OR mobile.ti,ab. OR app.ti,ab. OR web.ti,ab. OR web-based.ti,ab. OR online.ti,ab. OR phone.ti,ab. OR internet.ti,ab. OR "internet-based cognitive behavioral therapy".ti,ab. OR "web-based cognitive behavioral therapy".ti,ab. OR "online cognitive behavioral therapy".ti,ab. OR "digital cognitive behavioral therapy".ti,ab.) AND ("cardiovascular disease".ti,ab. OR "heart disease".ti,ab. OR "coronary heart disease".ti,ab. OR "coronary artery disease".ti,ab. OR "ischemic heart disease".ti,ab. OR "heart attack".ti,ab. OR "heart failure".ti,ab. OR "cardiac failure".ti,ab. OR "acute coronary syndrome".ti,ab. OR "myocardial infarction".ti,ab. OR "peripheral arterial disease".ti,ab. OR "peripheral occlusive disease".ti,ab.) AND ("cardiac rehabilitation".ti,ab. OR "secondary prevention".ti,ab. OR "stress management".ti,ab. OR "stress reduction".ti,ab. OR distress.ti,ab. OR stress.ti,ab. OR self-management.ti,ab. OR self-efficacy.ti,ab. OR "quality of life".ti,ab. OR "risk factor modification".ti,ab. OR "risk factor reduction".ti,ab.) AND ("randomized controlled study".ti,ab. OR "randomized controlled trial".ti,ab.)<br>*(Polyglot [1] was used to convert the search term)*	41

**5**	Web of Science<br>15.11.2023	*Suchfeld "topic" für title, abstract und keyword:*<br>(eHealth OR e-Health OR mHealth OR telehealth OR "digital health intervention" OR mobile OR app OR web OR web-based OR online OR phone OR internet OR "internet-based cognitive behavioral therapy" OR "web-based cognitive behavioral therapy" OR "online cognitive behavioral therapy" OR "digital cognitive behavioral therapy") AND ("cardiovascular disease" OR "heart disease" OR "coronary heart disease" OR "coronary artery disease" OR "ischemic heart disease" OR "heart attack" OR "heart failure" OR "cardiac failure" OR "acute coronary syndrome" OR "myocardial infarction" OR "peripheral arterial disease" OR "peripheral occlusive disease") AND ("cardiac rehabilitation" OR "secondary prevention" OR "stress management" OR "stress reduction" OR distress OR stress OR self-management OR self-efficacy OR "quality of life" OR "risk factor modification" OR "risk factor reduction") AND ("randomized controlled study" OR "randomized controlled trial")<br><br>*(Polyglot [1] was used to convert the search term)*	540
**6**	ClinicalTrials.gov<br>15.11.2023	*Search field "Condition or disease":*<br>("cardiovascular disease" OR "heart disease" OR "coronary heart disease" OR "coronary artery disease" OR "ischemic heart disease" OR "heart attack" OR "heart failure" OR "cardiac failure" OR "acute coronary syndrome" OR "myocardial infarction" OR "peripheral arterial disease" OR "peripheral occlusive disease")<br><br>*Search field "Intervention/Treatment":*<br>(eHealth OR e-Health OR mHealth OR telehealth OR "digital health intervention" OR mobile OR app OR web OR web-based OR online OR phone OR internet OR "internet-based cognitive behavioral therapy" OR "web-based cognitive behavioral therapy" OR "online cognitive	280

		behavioral therapy" OR "digital cognitive behavioral therapy")<br><br><u>*Search field "Other terms":*</u><br>("cardiac rehabilitation" OR "secondary prevention" OR "stress management" OR "stress reduction" OR distress OR stress OR self-management OR self-efficacy OR "quality of life" OR "risk factor modification" OR "risk factor reduction") AND ("randomized controlled study" OR "randomized controlled trial")	
**7**	German Clinical Trials Register (GermanCTR) 15.11.2023	(eHealth OR e-Health OR mHealth OR telehealth OR "digital health intervention" OR mobile OR app OR web OR web-based OR online OR phone OR internet OR "internet-based cognitive behavioral therapy" OR "web-based cognitive behavioral therapy" OR "online cognitive behavioral therapy" OR "digital cognitive behavioral therapy") AND ("cardiovascular disease" OR "heart disease" OR "coronary heart disease" OR "coronary artery disease" OR "ischemic heart disease" OR "heart attack" OR "heart failure" OR "cardiac failure" OR "acute coronary syndrome" OR "myocardial infarction" OR "peripheral arterial disease" OR "peripheral occlusive disease") AND ("cardiac rehabilitation" OR "secondary prevention" OR "stress management" OR "stress reduction" OR distress OR stress OR self-management OR self-efficacy OR "quality of life" OR "risk factor modification" OR "risk factor reduction") AND ("randomized controlled study" OR "randomized controlled trial")	25
**8**	International Clinical Trials Registry Platform (ICTRP) 15.11.2023	(eHealth OR e-Health OR mHealth OR telehealth OR "digital health intervention" OR mobile OR app OR web OR web-based OR online OR phone OR internet OR "internet-based cognitive behavioral therapy" OR "web-based cognitive behavioral therapy" OR "online cognitive behavioral therapy" OR "digital cognitive behavioral therapy") AND ("cardiovascular disease" OR "heart disease" OR "coronary heart disease" OR "coronary artery disease" OR "ischemic heart disease" OR "heart attack" OR	28

		"heart failure" OR "cardiac failure" OR "acute coronary syndrome" OR "myocardial infarction" OR "peripheral arterial disease" OR "peripheral occlusive disease") AND ("cardiac rehabilitation" OR "secondary prevention" OR "stress management" OR "stress reduction" OR distress OR stress OR self-management OR self-efficacy OR "quality of life" OR "risk factor modification" OR "risk factor reduction") AND ("randomized controlled study" OR "randomized controlled trial")	
**9**	(International Standard Randomised Controlled Trial Number Registry (ISRCTN registry) 15.11.2023	(eHealth OR e-Health OR mHealth OR telehealth OR "digital health intervention" OR mobile OR app OR web OR web-based OR online OR phone OR internet OR "internet-based cognitive behavioral therapy" OR "web-based cognitive behavioral therapy" OR "online cognitive behavioral therapy" OR "digital cognitive behavioral therapy") AND ("cardiovascular disease" OR "heart disease" OR "coronary heart disease" OR "coronary artery disease" OR "ischemic heart disease" OR "heart attack" OR "heart failure" OR "cardiac failure" OR "acute coronary syndrome" OR "myocardial infarction" OR "peripheral arterial disease" OR "peripheral occlusive disease") AND ("cardiac rehabilitation" OR "secondary prevention" OR "stress management" OR "stress reduction" OR distress OR stress OR self-management OR self-efficacy OR "quality of life" OR "risk factor modification" OR "risk factor reduction") AND ("randomized controlled study" OR "randomized controlled trial")	204
**Total hits**			**4.078**
**Total hits without duplicates**			**2.990**

Results

Meta-analysis

The meta-analyses were carried out for the different outcome parameters on the basis of a total of seven study reports [2-8].

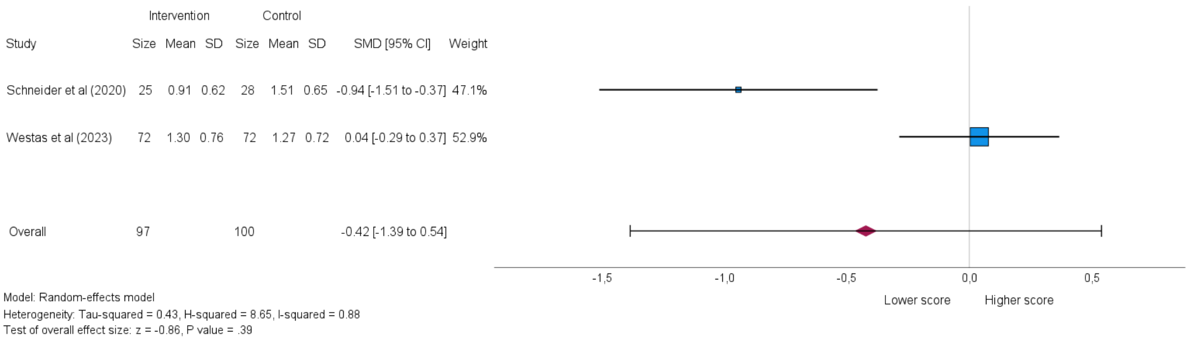

Figure S1. Forest plot showing the effect of eHealth stress management interventions on the CAQ-Attention score among patients with cardiovascular diseases. CAQ = Cardiac Anxiety Questionnaire; SD = standard deviation, SMD = standard mean difference, CI = confidence interval

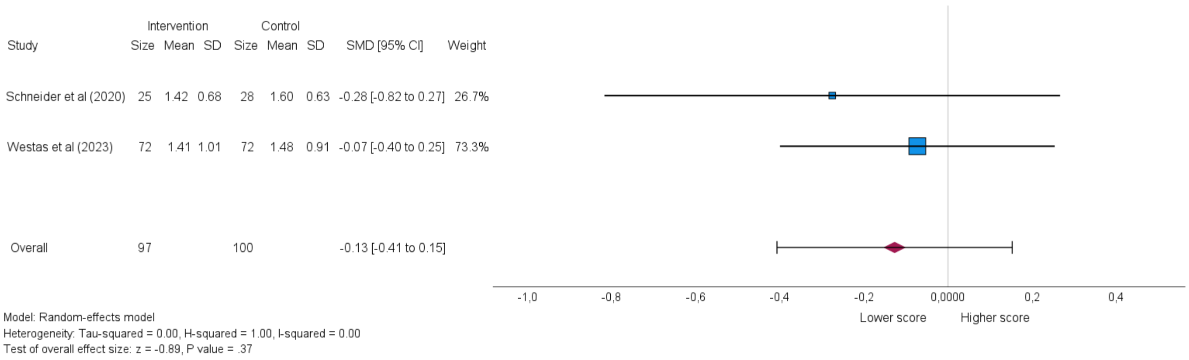

Figure S2. Forest plot presenting the impact of eHealth stress management interventions on the CAQ-Avoidance score among patients with cardiovascular diseases. CAQ = Cardiac Anxiety Questionnaire; SD = standard deviation, SMD = standard mean difference, CI = confidence interval

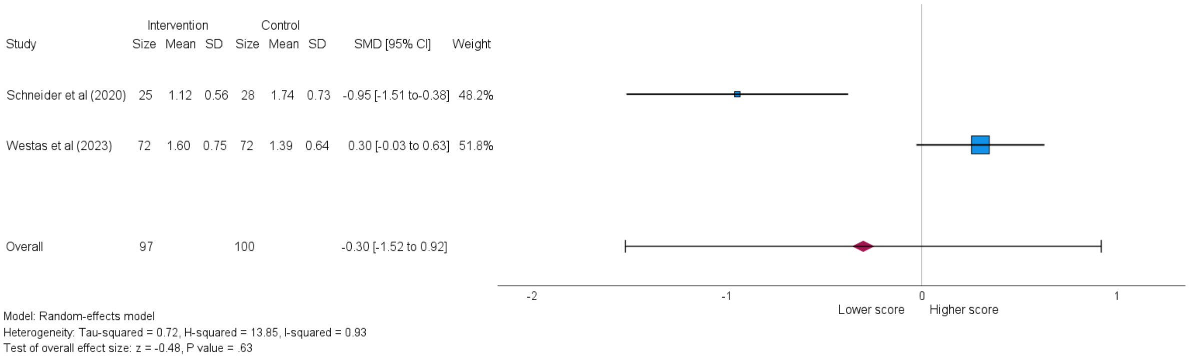

Figure S3. Forest plot showing the effect of eHealth stress management interventions on the CAQ-Fear score among patients with cardiovascular diseases. CAQ = Cardiac Anxiety Questionnaire; SD = standard deviation, SMD = standard mean difference, CI = confidence interval

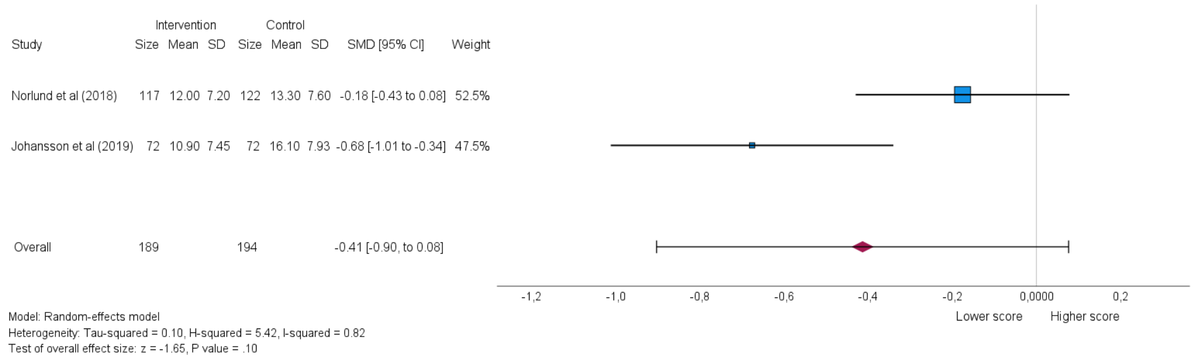

Figure S4. Forest plot showing the effect of eHealth stress management interventions on MADRS-S score in patients with cardiovascular diseases. MADRS-S =; Montgomery–Åsberg Depression Rating Scale-Self Assessment SD = standard deviation, SMD = standard mean difference, CI = confidence interval

Sensitivity analysis

Table S2. Findings of the conducted sensitivity analysis

Outcome	Mean Difference [95% CI]	Standard Error	P Value	I²
CAQ-Total	-0.20 [-0.77 to 0.37]	0.29	.49	0.90
CAQ-Attention	-0.27 [-0.89 to 0.34]	0.31	.39	0.88
CAQ-Avoidance	-0.12 [-0.35 to 0.12]	0.12	.32	0.00
CAQ-Fear	-0.19 [-1.01 to0.62]	0.41	.64	0.93
GAD-7	-2.64 [-5.96 to 0.67]	1.69	.12	0.86
MADRS-S	-3.16 [0.11 to -6.98]	1.95	.11	0.83
PHQ-9	-2.33 [-3.35 to -1.31]	0.52	<.001	0.12
SF-12 Physical Health	1.72 [-1.20 to 4.64]	1.49	.25	0.47
SF-12 Mental Health	3.89 [1.60 to 6.18]	1.17	<.001	0.00

References

1. Clark JM, Sanders S, Carter M, Honeyman D, Cleo G, Auld Y, et al. Improving the translation of search strategies using the Polyglot Search Translator: a randomized controlled trial. J Med Libr Assoc. 2020 Apr;108(2):195-207. PMID: 32256231. doi: 10.5195/jmla.2020.834.
2. Bendig E, Bauereiss N, Buntrock C, Habibovic M, Ebert DD, Baumeister H. Lessons learned from an attempted randomized-controlled feasibility trial on “WIDeCAD” - An internet-based depression treatment for people living with coronary artery disease (CAD). Internet Interv. 2021;24:100375. PMID: CN-02267051. doi: 10.1016/j.invent.2021.100375.
3. O'Neil A, Taylor B, Sanderson K, Cyril S, Chan B, Hawkes AL, et al. Efficacy and feasibility of a tele-health intervention for acute coronary syndrome patients with depression: results of the "MoodCare" randomized controlled trial. Ann Behav Med. 2014 Oct;48(2):163-74. PMID: 24570217. doi: 10.1007/s12160-014-9592-0.
4. Johansson P, Westas M, Andersson G, Alehagen U, Broström A, Jaarsma T, et al. An Internet-Based Cognitive Behavioral Therapy Program Adapted to Patients With Cardiovascular Disease and Depression: Randomized Controlled Trial. JMIR Ment Health. 2019 Oct;6(10):e14648. PMID: 31584000. doi: 10.2196/14648.
5. Lundgren JG, Dahlström Ö, Andersson G, Jaarsma T, Kärner Köhler A, Johansson P. The Effect of Guided Web-Based Cognitive Behavioral Therapy on Patients With Depressive Symptoms and Heart Failure: A Pilot Randomized Controlled Trial. J Med Internet Res. 2016 Aug;18(8):e194. PMID: 27489077. doi: 10.2196/jmir.5556.
6. Westas M, Mourad G, Andersson G, Lundgren J, Johansson P. The effects of internet-based cognitive behavior therapy for depression in cardiovascular disease on symptoms of anxiety: A secondary analysis of a randomized trial. Eur J Cardiovasc Nurs. 2024 Sep;23(4):382-90. PMID: 37740442. doi: 10.1093/eurjcn/zvad097.
7. Schneider LH, Hadjistavropoulos HD, Dear BF, Titov N. Efficacy of internet-delivered cognitive behavioural therapy following an acute coronary event: A randomized controlled trial. Internet Interv. 2020 Sep;21:100324. PMID: 32455120. doi: 10.1016/j.invent.2020.100324.
8. Norlund F, Wallin E, Olsson EMG, Wallert J, Burell G, von Essen L, et al. Internet-Based Cognitive Behavioral Therapy for Symptoms of Depression and Anxiety Among Patients With a Recent Myocardial Infarction: The U-CARE Heart Randomized Controlled Trial. J Med Internet Res. 2018 Mar;20(3):e88. PMID: 29519777. doi: 10.2196/jmir.9710.
